# Novel cause of Acute Kidney Injury and its Predictors of Mortality

**DOI:** 10.12669/pjms.42.4.14130

**Published:** 2026-04

**Authors:** Rubina Naqvi, Zara Khan

**Affiliations:** 1 Rubina Naqvi, Department of Nephrology, Sindh Institute of Urology and Transplantation (SIUT), Karachi - 74200, Pakistan; 2 Zara Khan, Department of Nephrology, Sindh Institute of Urology and Transplantation (SIUT), Karachi - 74200, Pakistan

**Keywords:** Acute Kidney Injury (AKI), Acute Gastro-enteritis (AGE), Acute Diarrhea, Mortality predictors, Renal Replacement Therapy (RRT)

## Abstract

**Objective:**

Hypovolemia resulting from loss of massive amount of fluids via gastro intestinal tract and causing acute kidney injury (AKI) is seen less in developed countries, while in developing countries we still see large number of such cases. Present study is largest cohort of cases developing hypovolemia from gastro intestinal fluid loss causing AKI and study highlights mortality predictors related to this scenario.

**Methodology:**

It is a retrospective cohort where case records of all the patients between January 1990 to December 2024, coming to Sindh Institute of Urology and Transplantation (SIUT), Karachi; who were labelled to have AKI according to KDIGO criteria, were reviewed. Patients developing AKI after acute diarrheal illness, with or without vomiting, were included and the data was analyzed and results are presented here.

**Results:**

During the specified time of 35 years, total 1,456 cases of AKI after acute diarrheal illness were seen at this institution, these were 15.2% of total AKI. Mean age was 39.36 ±17.10(range from 18-100 years), male to female ratio was 2.21:1. Non-oliguric AKI was seen in 422 (29%), while 889 (61%) were oliguric and 145 (10%) were anuric. Renal replacement therapy in form of hemodialysis was required in 959 (66%) patients, while 497 (34%) were managed conservatively. Complete renal recovery was seen in 1160 (79.67%), while 125 during (8.58%) died acute phase of illness. Mortality was higher in patients who were hypotensive requiring inotropes, required mechanical ventilation, presented in altered state of consciousness, or developed cardiac arrhythmias with severe electrolyte imbalance. Many of those who died had combination of mentioned insults.

**Conclusion:**

While world is moving towards digital and revolutionizing renal diagnosis globally, we face a reality of too many patients suffering from AKI with a preventable cause. We are losing lives with causes where AKI should not occur in first place.

## INTRODUCTION

Acute Kidney Injury (AKI), is the sudden loss of kidney functions which may be mild and rapidly reversible to severe enough to require renal replacement therapy and sometimes may not reverse. This irreversibility depends on many factors ranging from cause to the duration of insult happened and the scenario where other insults add-on to primary cause. AKI which previously called as Acute Renal Failure (ARF) was lacking a standard definition for decades and its categorization varied from center to center. Then some criteria known as RIFLE established in 2002, then AKIN in 2005 and most recently in use is KDIGO criteria for defining AKI.^1^ The true incidence of AKI has wide variation among reports from developing and developed countries and so lies the difference among causes. The incidence is reported as 22% in the high-income countries or developed world and 7.5% in lower and lower-middle-income countries or developing world.^2^ Pre-renal causes of AKI are rare to see in high income countries where AKI mostly seen in intensive care setting in context of multi organ failure, or after extensive surgeries or trauma or sepsis.^3^ Whereas, in developing world we still see such patients in large numbers; for example volume loss around child birth and pregnancy related AKI reported in 25.6% of total AKI in a large published series from country. The same study reports pre renal AKI from volume loss during acute gastro enteritis as 15% of total AKI population.^4^

The kidneys are receiving up to 25% of the cardiac output,^5^ any factor reducing the circulatory volume and abating the compensation, results in sudden ischemic injury causing AKI. Sudden and massive loss of fluids from gastro-intestinal tract, as with acute diarrhea and/or intractable vomiting are such scenarios. AKI after acute gastro-enteritis (AGE) is a rare entity heard in developed world, a systematic review comprising literature from high income countries is about pediatric population where diarrhea in most studies was associated with hemolytic uremic syndrome.^6^ Reports from our part of the world vary from 13.6% to 60.62% of AGE patients develop AKI.^7^,^8^

In a case series published from India, they have reported six cases of AKI developing after acute gastro enteritis over a span of three months and 50% of these required renal replacement therapy in form of hemodialysis, ranging from 4-12 sessions.^9^ Another study from India reported handling 101 acute gastro enteritis patient over a span of 5 months and 52% of these developed AKI according to KDIGO criteria, while 38% of the AKI were in stage 1, still 19% were progressed to stage requiring HD, 75% in this study show complete renal recovery.^10^

Our previously published community acquired AKI have revealed AGE contributing 15% of total AKI, now with further expansion of work and managing AKI for a total of 35 years we have largest cohort of AKI secondary to AGE, ever reported globally.

## METHODOLOGY

It is a retrospective cohort where case records of all the patients between January 1990 to December 2024, coming to Sindh Institute of Urology and Transplantation (SIUT), Karachi; with a diagnosis of AKI were reviewed. AKI was defined by KDIGO criteria. Patients who developed AKI after acute gastro-enteritis were included in present cohort, while other causes of AKI, patients with systemic diseases like long standing hypertension or diabetes mellitus or renal stone disease were excluded.

### Ethical Approval:

Proposal was submitted to Ethics Review Committee (ERC) of SIUT before starting data collection and approval was granted with Number SIUT-ERC/2020/A-216; dated: August 10, 2020.

Volume replacement was first strategy of management in those who still had urine output. While those who get transferred from other hospitals and already had fluid replacement challenge or those who had oliguria (urine output <400 mL/day) or anuria (urine output <100mL/day) were started on renal replacement therapy (RRT) in form of hemodialysis.

The complete recovery was labelled when patients achieved and sustained serum creatinine levels <1 mg/dl, while partial recovery was serum creatinine still >1 mg/dl but RRT no more required.

Case records from institutional record system were collected and parameters were recorded for all adult patients with above mentioned inclusion criteria. Recorded parameters included gender, age, duration of illness, decline in urine output (oliguria/ anuria), hematology; including hemoglobin, total white cells count, platelets count, prothrombin, activated partial thromboplastin time, biochemistry; including urea, creatinine, sodium, potassium, bicarb, calcium, albumin, lactate dehydrogenase, liver function tests and urinalysis. Stool analysis and microbiology cultures from stool, urine and blood in selected cases.

All data was entered on Excel sheet and mean, standard deviation and median were calculated on same.

## RESULTS

Between January 1990 - December 2024 (35 years), total 1,456 cases of AKI after acute diarrheal illness were seen at this institution, these are 15.2% of total AKI patients seen during this period. Male to female ratio was 2.21:1 (male=1001, female=451) in this population. Demographics and laboratory test on arrival to this institution are given in Table-I, where days of insult means the time from development of first symptom to time to reach to this institution. AKI was non-oliguric in 422 (29%), while 889 (61%) were oliguric and 145 (10%) were anuric. Seasonal variation was like 63% patients arrived between the months of April to September, while 37% between October to march. Renal replacement therapy in form of hemodialysis was required in 959 (66%) patients. Parenteral fluids were given to all except 31 patients who presented with absolute anuria. Complete renal recovery was seen in 1160 (79.67%) patients, of these 454 were only managed conservatively (RRT not required), 125 (8.58%) died during acute phase of illness. Ten patients when suggested for dialysis left against medical advice, 113 (7.76 %) remained long term follow up in partial state of renal recovery, while four patients required lifelong RRT. The remaining 44 (3.02%) patients did not turn up for follow up after discharge from hospital. Predictors for mortality given in Table-II. More than one factor among listed complications were found in many deceased persons.

**Table-I:** Demography and baseline laboratory parameters (n=1,456).

Parameter	Mean ±std	Median	Range
Age (years)	39.36 ±17.10	35	18-100
Days of Insult	6.36 ± 3.74	5	1-20
Hemoglobin (11.5-15.4 G/dl)	12.94 ± 2.68	12.9	3.1-22
WCC (4-11 x10^3^/μl)	15.24 ± 7.55	13.4	1.5-71.8
Platelet (150-400 x10^3^/μl)	260.94 ± 119.30	240	14-1038
Urea (15-39 mg/dl)	221.06 ± 118.39	198	41-686
Creatinine (0.5-1.2 mg/dl)	8.92 ± 4.42	8.2	1.6 -41.7
Na (135-145 mmol/L) <135= 931, >145=55	131.84 ± 8.42	132	84-171
K (3.5-5.0 mmol/L) >5 = 133, <3.5 =639	5.44 ± 0.85	5.2	1.2-9.3
HCO3 (22-30 mmol/L) >30 = 6, <22 =1374	11.96 ± 5.41	11	4-48
LDH (140-280 U/L)	585.59 ± 549.36	408	25-5490
Calcium (8.5-10.2 mg/dL) >10.2 = 172 <8.5 =592	8.71 ± 1.49	8.4	3.8-15.1
Albumin (3.5-5.5 g/DL) >5.5 = 70 <3.5 =557	3.66 ± 1.06	3.5	1.2-10.2
SGOT /AST (8-45 U/L)	82.58 ± 255.53	43	8 – 6280
SGPT/ ALT (7-56 U/L)	74.17 ± 277.10	35	4-5040

**Table-II:** Predictors for mortality (n=125).

Clinical Status	Number of patients
Altered state of consciousness	64
Required mechanical ventilation	46
Presented with hypotension (Inotropes required)	25
Arrhythmias	20
Pulmonary edema	06
GI Bleed	04
More than one of the above	48

## DISCUSSION

The patients with acute gastro-enteritis or acute diarrhea are common visitors of any emergency department of state or private hospitals. They are usually managed there with parenteral fluids and antibiotics, irrespective of fact that acute diarrhea in many cases could be viral origin. Norovirus; a single stranded RNA virus from Caliciviridae family, is common causative virus for acute gastroenteritis, it is highly contagious as well. Rotavirus; another virus causing acute gastro-enteritis is more commonly affects the children. Among bacterial AGE; which can affect single person or group of people eating together, organisms included E.coli, Compylobacter jijuni, Salmonella, Shigella, S.aureus, Yersinia, Vibrio cholera etc.

Acute kidney injury is a serious complication of AGE, while it can be prevented if fluid loss is replaced timely and sensibly, when occurs it causes prolonged hospitalizations, referral to nephrology care units, increased morbidity, high cost at management and negative long-term consequences. We at a tertiary care renal unit often receive patients with advanced uremia, electrolyte imbalances, acidosis. Many of our studied patients with marked decline in urine output already received ample parenteral fluids that some of them land here with pulmonary edema and require mechanical ventilator support at arrival.

The published literature from developed world, on AKI after AGE is scarce an Italian study only reports pediatric population.^11^ From India, Pakistan, Saudi Arabia and Afghanistan published articles describe limited number of adult patients.^7^-^10^,^12^ Massive volume loss with severe diarrhea leads to hemo-concentration which was reflected from high hemoglobin and hematocrit values in our studied population 334 (23%), irrespective of gender had hemoglobin more than 15 G/dl on presentation. This was also reflected in our population with high serum albumin and serum calcium levels in notable number of our population Table-I. Electrolyte imbalance was also frequent observation severe hypokalemia with serum potassium less than two was seen in 31 patients while severe hyperkalemia with levels more than 6 in 40 patients.

Overall below normal potassium level was found in 639 (44%) and above normal in 133 (9%) Table-I. But either extreme were risk for cardiac arrhythmias which was one of predictor for mortality. Similarly, severe hypo or hypernatremia was contributing to altered level of consciousness which is another predictor for mortality Table-II. Other published studies have not mentioned such wide variations in serum potassium levels,^8^,^12^ though Boghari et al. reported high potassium level but not statistically significant when compared with AGE without AKI.^7^ Aggressive parenteral fluid therapy, at primary health care, with overlooking urine output resulted in pulmonary edema and electrolyte imbalance. One of the patient has infused 38L of intra-venous fluid over a span of 42 hours before shifting to this institution.

Some of our studied population had blood in stools and presented with very low hemoglobin levels, 3.1 g/dl was the lowest we received. Bleeding from gastro intestinal tract was also mortality predictor. Patients reaching with hypovolemic shock and low urinary output required inotropic support, many of these and others with pulmonary edema required mechanical ventilator support on arrival and could not succumb.

Medical data base search engines lacking in support of AKI secondary to AGE in adult population, a case report published from Australia describes 65-year male developing AKI along with multi-organ failure after Rota virus acute gastro-enteritis.^13^

Most of published literature on AKI after AGE addresses the pediatric population. A recent published study from country, on pediatric population describes AKI occurring in 20.75% of children hospitalized with severe diarrhea.^14^ Whereas, another recent study from country, again in pediatric population found AKI occurring in 8.6% of children hospitalized with diarrhea.^15^

In summary, while reporting largest cohort of AKI secondary to AGE, to date, our findings confirm that this condition poses a year around risk and it is not confined to any season, though summer months have higher numbers than winter (63 vs 37%). Early addressing volume loss and at the same time meticulous watch over urine output and serum electrolytes is a must which should be strictly done by primary physician. There should be no hesitation to refer the patient to a facility where a check on these can be better observed and managed.

### Limitations:

The study has limitations of being a retrospective design and the available information is all that was recorded at that time.

## CONCLUSION

It is important to report a “novel”, which in its actual meaning is not new, but unexpected in a way that in developed world no one expects such a high number of patients developing AKI after AGE. The medical community should be aware of seriousness and timely addressing the management of a simple yet dangerous life threatening disease entity. There should be awareness campaigns introduced to educate health care workers at basic health units to identify, take prompt steps in management and timely refer these patients to place where treating facilities for such patients are available. This can avoid further deterioration and can cut down mortality rate.
